# Systemic Lupus Erythematosus (SLE) and Antineutrophil Cytoplasmic Antibody-Associated Vasculitis (AAV) Overlap Syndrome: Case Report and Review of the Literature

**DOI:** 10.1155/2019/5013904

**Published:** 2019-01-06

**Authors:** Sathish Itikyala, Debendra Pattanaik, Syed Raza

**Affiliations:** University of Tennessee Health Science Center, Division of Rheumatology, Memphis, TN, USA

## Abstract

We report here the first case of systemic lupus erythematosus (SLE) and antineutrophil cytoplasmic antibody-associated vasculitis overlap syndrome (SLE/AAV) who had granulomatous polyangiitis (GPA) as the initial presentation. SLE/AAV overlap syndrome is an uncommon entity recently described in the literature. Prior reported patients with SLE/AAV overlap syndrome presented with SLE and microscopic polyangiitis (MPA). Our patient initially presented with granulomatous gastric ulcer and later developed respiratory failure. She was diagnosed with GPA. While on maintenance treatment with azathioprine 150 mg/day, she developed hematuria and proteinuria which turned out to be from class V lupus nephritis instead of relapse of vasculitis. Currently, the patient is doing well after treatment with rituximab. Although rare, this entity should be recognized and need to be treated appropriately.

## 1. Introduction

Systemic lupus erythematosus (SLE) is a multisystem autoimmune disease characterized by the presence of autoantibodies and heterogeneous clinical presentation [1]. Antineutrophil cytoplasmic antibody- (ANCA)-associated vasculitis (AAV) on the other hand is a necrotizing vasculitis involving small to medium vessels and presence of antineutrophil cytoplasmic antibodies (ANCAs) specific for either myeloperoxidase (MPO) or proteinase 3 (PR3) [2]. AAV includes three major clinicopathologic variants: granulomatosis with polyangiitis (GPA, Wegener's granulomatosis), microscopic polyangiitis (MPA), and eosinophilic granulomatosis with polyangiitis (EGPA, Churg–Strauss syndrome). The prevalence of ANCAs is reported to be as high as 31% in lupus patients but its role in SLE has no demonstrable clinical significance [3]. SLE/AAV overlap syndrome is a recently described entity where subjects had SLE and microscopic polyangiitis with rapidly developing glomerulonephritis [4, 5]. Majority of the patients were diagnosed concomitantly with SLE and AAV [5]. Ninety-two percent of the reported cases presented with serious manifestations, e.g., rapidly progressive glomerulonephritis, and were treated aggressively with corticosteroids and cyclophosphamide [5]. Other common organ-threatening manifestations reported include CNS vasculitis and thrombotic cerebrovascular events [6]. Thirteen percent of the reported cases died of complications [5]. Our patient initially presented with PR3 positive AAV with involvement of the GI tract and lungs who eventually developed class V lupus nephritis. All the previously reported cases of SLE/AAV syndrome had AAV with + P-ANCA and MPO antibody, and most common renal manifestation was rapidly progressive glomerulonephritis unlike our case [5–8].

## 2. Case Presentation

**Figure 1:**
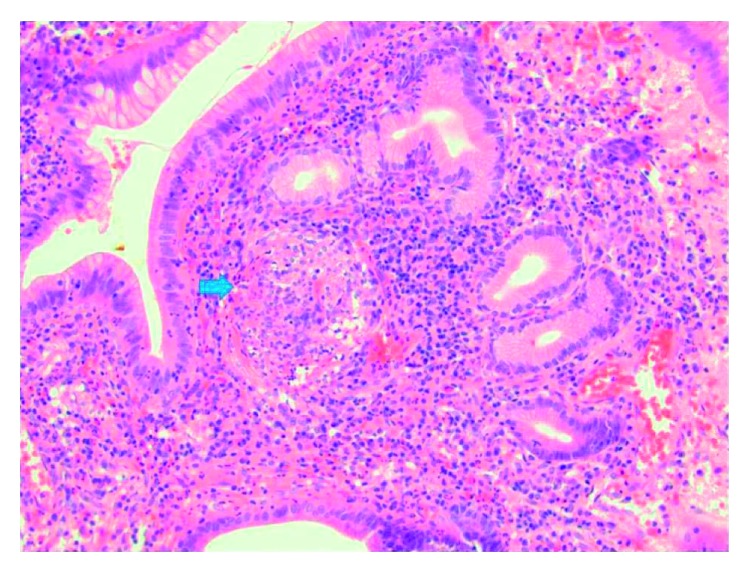
Gastric biopsy showing chronic gastritis with lymphoid aggregates and nonnecrotizing granulomatous inflammation with multinucleated giant cells.

A 48 -year-old Indian woman presented to the gastroenterology clinic with complaints of upper abdominal pain, heartburn, and unintentional 10-pound weight loss over a period of 6 months. Review of the system was negative for headache, nasal discharge, cough, chest pain, shortness of breath, sinusitis, joint pain, or skin rash. Past medical history is notable for hypothyroidism only. Family history and personal history is otherwise unremarkable. Her medications include levothyroxine 50 mcg daily. She underwent EGD, which revealed a solitary 1.4 cm ulcer in the gastric fundus. Biopsy of the ulcer revealed active chronic gastritis with lymphoid aggregates and nonnecrotizing granulomatous inflammation with multinucleated giant cells (Figure 1).

**Figure 2:**
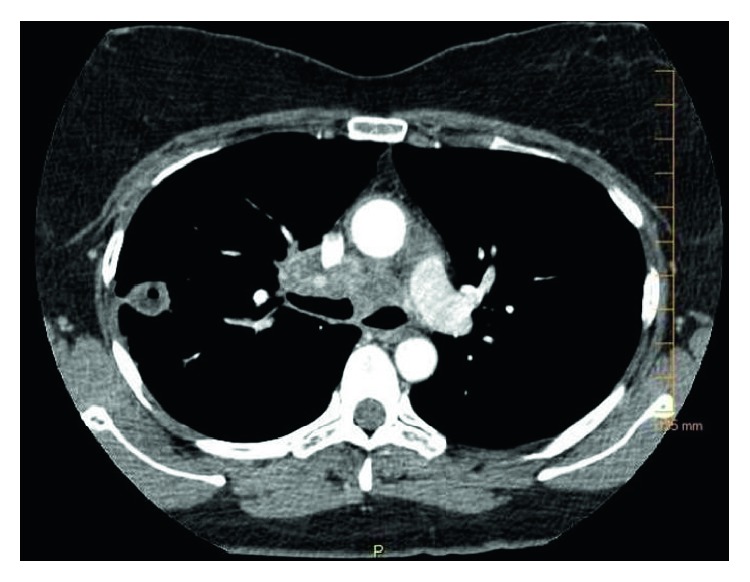
CT chest showing large necrotic mass.

Histochemical staining and culture results were negative for any bacterial, viral, and fungal infections. The biopsy was negative for gastric carcinoma. She was then empirically treated with proton pump inhibitors. Two months later, the patient presented to emergency room with cough, pleuritic chest pain, and hemoptysis. The CT chest showed two large necrotic masses (Figure 2): one in the right upper lobe with a cavitary lesion and the other in the mediastinum.

She eventually underwent elective bronchoscopy with transbronchial biopsies and bronchoalveolar lavage (BAL). Right upper lobe transbronchial biopsy showed bronchial mucosa with acute and chronic inflammation (lymphocytes, neutrophils, eosinophils, and rare histiocytes) and small submucosal microabscess. Lung alveolar parenchyma was without significant histopathological changes. The biopsy was negative for any form of lung malignancy. The BAL was negative for pulmonary hemorrhage. No fungi, pneumocystis, or viral inclusion bodies were identified in BAL. Bacterial and fungal and mycobacterial cultures of BAL were negative as well. Infectious disease workup showed positive histoplasma serum antibody but histoplasma urine antigen was negative. Presumed diagnosis of histoplasmosis was made, and she was started on oral itraconazole as outpatient therapy. The patient did not respond well, and her clinical status continued to decline. She then presented to the emergency room with spiking fevers and recurrence of hemoptysis. She was admitted to the inpatient service and started on IV amphotericin-B per infectious disease service recommendations. During hospitalization, she complained of epigastric discomfort. CT abdomen showed opacity around the previously found gastric ulcer and fluid collection adjacent to the stomach and pancreatic bed. Repeat EGD showed further development of a sinus tract in the ulcer, leaking exudate, and necrotic debris. Biopsies of the ulcer again showed mild active chronic gastritis. No evidence of infection or malignancy was identified. During the hospital course, she developed arthralgia and maculopapular pruritic skin lesions on the right flank and both legs. After a week into the hospitalization, the patient developed severe shortness of breath and acute respiratory failure that required intubation and mechanical ventilation. CT chest revealed worsening of the previous right upper lobe cavitary lesion with pleural effusion. Skin biopsy showed leukocytoclastic vasculitis. Further workup showed positive ANA in the low titer (1 : 40, homogenous pattern). Specific antibodies, e.g., anti-dsDNA, anti-Smith ab, anti-SSA and SS-B, were negative. Complements, C3: 113 (90–180 mg/dl) and C4: 12 (10–40 mg/dl), were within normal limits. The C-reactive protein level was 86 mg/dl (normal < 0.5 mg/dl). Serum protein electrophoresis showed polyclonal acute phase reactant pattern. C-ANCA was positive with positive PR3: 7.21 (0.8–1.19 AU/mL). Anti-MPO antibody was negative. At this point, the diagnosis of GPA was established.

**Figure 3:**
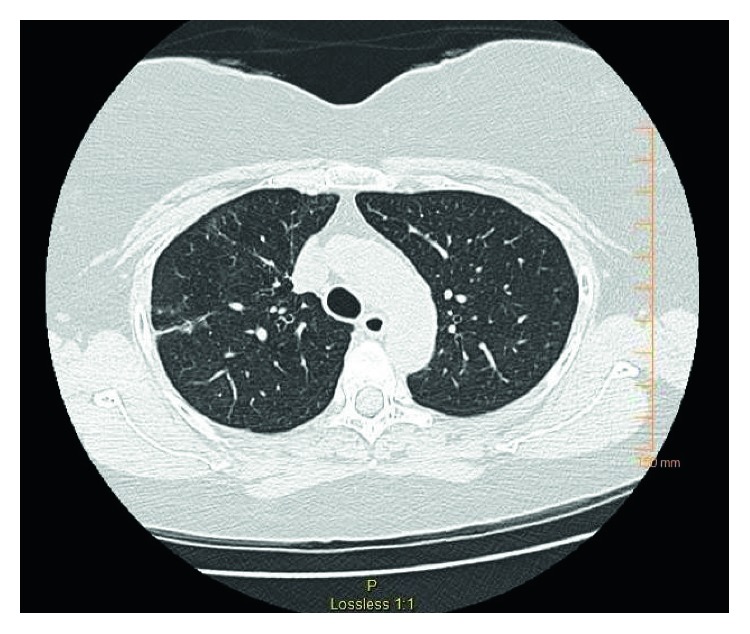
CT chest showing resolution of earlier chest lesion.

She was treated with pulse methylprednisolone 1 g IV daily for 3 days and IV cyclophosphamide 750 mg/m^2^. During hospitalization, she received a course of sulfamethoxazole-trimethoprim (800–160 mg DS) tab PO twice daily for 5 days for urinary tract infection. She responded very well and was promptly extubated and discharged in two weeks. She was maintained on PO prednisone 1 mg/kg/day and IV cyclophosphamide 750 mg/m^2^ monthly for the next 6 months. At 3 months post-hospital discharge follow-up, she was completely asymptomatic. Repeat anti-PR3 titer was 3.6 (normal 0.8–1.19 AU/mL). There was a near-complete resolution of pulmonary masses on repeat CT (Figure 3) and complete healing of the ulcer on follow-up EGD.

Prednisone was tapered to 10 mg/d over 6 months while she received monthly IV cyclophosphamide induction therapy. The PR3 antibody level was 1.0 and within normal limits. She was eventually started on PO methotrexate 15 mg/week as maintenance therapy for GPA. While she was on methotrexate, 6 months follow-up CT chest showed relapse of the necrotic lung lesions at the same place as before in the right upper lobe. The ANCA (PR-3) antibody level was 2.8 (normal 0.8–1.19 AU/mL). Clinically, she was asymptomatic. Again, she underwent extensive workup with bronchoscopy, and bronchoalveolar lavage was negative for any infection or malignancy. She was diagnosed with GPA relapse and treated with rituximab 375 mg/m^2^ every week for 4 weeks and was started on azathioprine 50 mg/day for maintenance therapy. Gradually, the azathioprine dose was increased to 150 mg/day over next 2 months. She was followed up as outpatient closely with repeat CT chest every 6 months, which showed regression of the lung lesion. Approximately, 2 years since her initial presentation, she was in complete remission with resolution of the lung lesion. Interestingly, about a year later, routine follow-up labs in the clinic revealed hematuria with 6 RBCs per high-power field (normal 0–4 per high-power field) and urine protein creatine ratio of 0.67 equivalent to 24 hours urinary protein of 670 mg. Follow-up 24-hour urine collection showed proteinuria of 1.3 gm. Physical examination was unremarkable. Her repeat antibody profile showed positive ANA: 206 (normal <100 au/ml), anti-dsDNA Ab:135 (normal <100 au/ml), anti-Smith Ab: 14 (normal < 100 au/ml), anti-RNP Ab: 36 (normal < 100 au/ml), SS-A Ab: 6 (normal < 100 au/ml), SS-B Ab: 7 (normal < 100 au/ml), antihistone Ab: 9 (normal < 100 au/ml), rheumatoid factor: 10 (normal < 14 au/ml), and anti-CCP Ab level: 1 (normal < 5 unit/ml). Follow-up complement levels were normal: C3, 152 (normal: 87–200 mg/dl), and C4, 25 (normal: 19–25 mg/dl). Repeat C-reactive protein levels were 0.6 and 0.7 (normal: < 5 mg/dl). Viral hepatitis screen and cryoglobulin screen were negative. Repeat ANCA and anti-PR3 as well as anti MPO antibodies were negative. Her antibody profile and proteinuria were concerning for new-onset SLE with renal involvement. She was then referred to nephrology and underwent renal biopsy.

**Figure 4:**
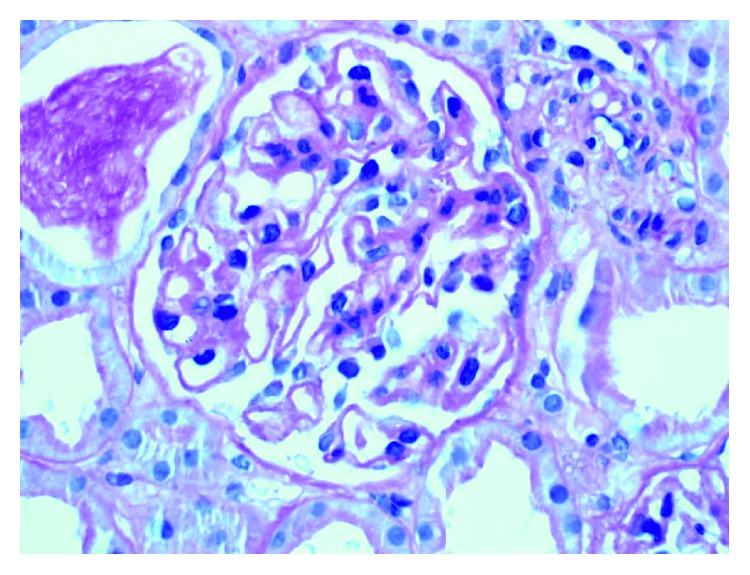
Renal biopsy (LM) showing diffuse thickening of the glomerular basement membrane with normal cellularity.

**Figure 5:**
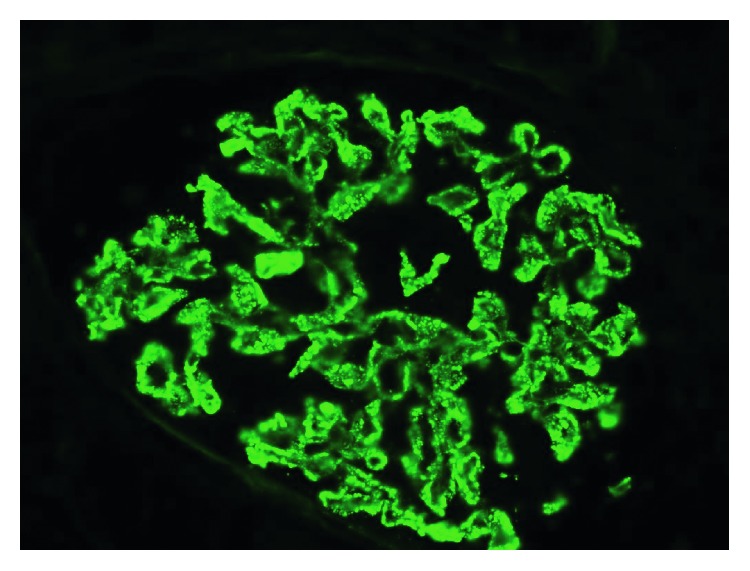
Renal biopsy (immunofluorescence microscopy) showing diffuse, granular immunoglobulin deposition along GBM.

**Figure 6:**
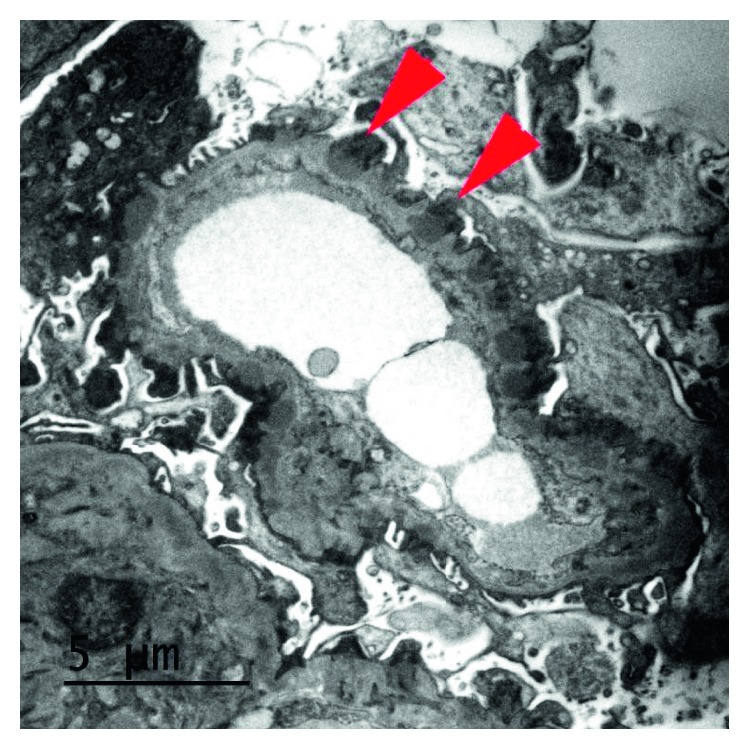
Renal biopsy (EM) showing subepithelial granular deposits.

Renal biopsy showed diffuse thickening of the glomerular basement membrane (GBM) with normal cellularity on light microscopy (Figure 4). Mesangial and endocapillary proliferation were negligible. No cellular crescents, pseudocrescents, or acute necrotizing lesions or vasculitis are seen. Immunofluorescent microscopy showed diffuse, granular immunoglobulin deposition along GBM (Figure 5). Immunostaining was positive for IgG, C3. Electron micrograph (Figure 6) showed subepithelial granular deposits but no subendothelial deposits. Congo red stain for amyloidosis was negative. Staining for anti-phospholipase A2 receptor (PLA2R) and anti-thrombospondin (THSD7a) antibodies were negative, which makes primary membranous nephropathy less likely. Considering the + ANA and double-stranded DNA Ab, the renal biopsy diagnosis was consistent with secondary pure membranous (type V) lupus nephritis. Ultimately, she was diagnosed with SLE/AAV overlap syndrome with class V membranous lupus nephropathy. She was started on lisinopril and was titrated to 20 mg/day. Azathioprine was slowly tapered and discontinued because she has finished 3 years of treatment for GPA. She declined high-dose steroid and mycophenolate mofetil for treatment of lupus nephritis. On follow-up CT scan, she had minor relapse of GPA in the form of enlargement of pulmonary nodule even though she was asymptomatic. The ANCA (PR-3) antibody titer was 1.7 (normal 0.8–1.19 AU/mL). She was given another round of rituximab 375 mg/m^2^ without induction corticosteroid dose. Follow-up 6 months CT chest showed resolution of the mass. Urinalysis and urine protein/creatinine ratio are negative for proteinuria and hematuria. She is completely asymptomatic and back to her normal activities. The patient has been in full remission for lupus nephritis for 1 year and for 9 months after the last relapse of GPA.

## 3. Discussion

Our patient was diagnosed with ANCA-associated vasculitis who had an unusual initial GI presentation that led to a delay in diagnosis. Subsequently, she developed respiratory failure and was treated with high-dose steroids and cyclophosphamide followed by methotrexate. Later on, she had a relapse which had to be treated with rituximab followed by maintenance azathioprine. She was treated with azathioprine because at that time, the role of maintenance rituximab therapy for GPA was not established. Three years from her initial presentation, she developed hematuria and significant proteinuria. The initial thought was that she was having a relapse of GPA who failed treatment with azathioprine which turned out to be not true. In fact, GPA was in remission. Given that she was asymptomatic otherwise and had negative repeat ANCA titer, we pursued other possible etiologies for her renal disease. Further laboratory testing showed positive lupus serologies and renal biopsy showed secondary membranous glomerulonephritis likely from SLE. It is unlikely that itraconazole, amphotericin-B, and sulfamethoxazole-trimethoprim would be responsible for lupus nephritis. The patient was compliant with azathioprine as she was extremely reliable, and her pharmacy record was verified that she was filling the prescription regularly. In fact, the GPA was in remission as well. The patient decided to try ACE inhibitor therapy instead of mycophenolate and prednisone for membranous lupus nephritis.

Systemic lupus erythematosus (SLE) is a chronic autoimmune disease mediated by autoantibody deposition in a variety of target organs, and AAV is a systemic vasculitis a mediated by antibodies targeting the granules in neutrophils, most commonly antimyeloperoxidase or antiproteinase [1, 2]. SLE and AAV share clinical features, such as arthritis, cutaneous lesions, as well as renal involvement [5]. Some patients meeting classification criteria for both SLE and AAV are categorized as SLE-AAV overlap syndrome [5]. Patients with SLE-AAV overlap syndrome are predominantly women, with a wide range of age distributions [5]. They usually present with arthritis, cytopenia, and renal involvement with necrotizing and crescentic glomerulonephritis and have positive ANA and anti-MPO antibodies [5]. In these cases, renal histopathology may help to distinguish ANCA vasculitis from SLE vasculitis [6]. The prevalence of ANCA is reported to be as high as 31% in lupus patients but its role in SLE pathogenesis has no demonstrable significance [3]. Vasculitis prevalence in SLE is reported to be between 11% and 36% which can involve vessels of all sizes, and it is generally mediated by complement and immune complex deposition [7, 9]. It predominantly involves small vessels with cutaneous lesions, and medium vessel vasculitis may present with visceral involvement and present with serious manifestations such as mesenteric vasculitis and pulmonary hemorrhage [9]. Lupus nephritis is an immune complex-mediated glomerulonephritis [10]. On the other hand, renal involvement in AAV is typically a pauci-immune crescentic glomerulonephritis [5]. Depending on the types of lupus nephritis, immune complexes are deposited predominantly in the mesangium and subendothelial or subepithelial sites [10]. Immune complex deposition in the glomeruli leads to activation of complement system, inflammatory cell infiltration, cytokine release, and cellular proliferation. In lupus nephritis, subendothelial deposits correlates with extent of endocapillary proliferation, and the endocapillary proliferations are associated with fibrinoid necrosis and crescent formation [8]. In AAV, activated neutrophils when primed by ANCAs release cytokines, lytic enzymes, and radical oxygen metabolites. These in turn damage endothelial cells and cause basement membrane necrosis, rupture, and crescent formation [11]. In contrast to LN, renal involvement in AAV is associated with paucity of immune complex deposition, absence of significant cellular proliferation, extensive fibrinoid necrosis, and crescent formation [8]. Finally, there is a subgroup of LN patients (class IV segmental and class III) who develop extensive segmental necrosis and crescent formation without subendothelial immune deposit or significant endocapillary proliferation but have positive P-ANCA and MPO antibodies [8, 12]. This lupus patient population has nephritis which resembles glomerulonephritis of ANCA-associated vasculitis [8, 12]. In SLE/AAV syndrome reported so far, renal pathology is classified either LN or pauci-immune GN according to the predominant type of lesion but some patients have overlapping features [5]. Majority (75–80%) patients have mesangial deposits and interstitial inflammation, 12% had subendothelial deposits, 50% had subepithelial deposits, and 40% had crescents/necrosis in glomeruli [5]. Our patient had pure class V lupus nephritis without any features of class IV LN, e.g., extensive endocapillary proliferation or subendothelial deposits or that of ANCA-associated vasculitis.

Our case is unique because the initial presentation was GPA with positive PR3 antibody followed by development of membranous lupus GN compared to what has been described in the literature regarding SLE-AAV overlap syndrome. She developed membranous GN and positive ANA and dsDNA antibodies while on azathioprine 150 mg/day over past 2 years. Development of lupus GN instead of pauci-immune GN from GPA was unexpected for a patient who previously had GPA and was receiving maintenance immunosuppressive therapy. The new finding changed the course of treatment, and the patient was treated for membranous lupus nephritis with ACE inhibitor rather than for relapse of GPA.

Our case expands the spectrum of SLE-ANCA overlap syndrome. Our case suggests that SLE-AAV overlap syndrome should be considered in the differential diagnosis of subjects who develop lupus or AAV with prior diagnosis of AAV or lupus, respectively. Renal biopsy should be considered in any AAV patients who develop abnormal urinary sediments instead of assuming possible AAV flare as this would lead to significant change in management.
